# Intermittent Theta Burst Transcranial Magnetic Stimulation Improves Language and Functional Connectivity in Primary Progressive Aphasia

**DOI:** 10.1002/alz70858_106722

**Published:** 2025-12-26

**Authors:** Thiago Paranhos, Anna Du, Nneka Watson, Daisy Hochberg, Megan Quimby, Neguine Rezaii, Bonnie Wong, Yuta Katsumi, Brad C. Dickerson, Mark C. Eldaief, Alexandra Touroutoglou

**Affiliations:** ^1^ Frontotemporal Disorders Unit, Department of Neurology, Massachusetts General Hospital and Harvard Medical School, Boston, MA, USA; ^2^ Frontotemporal Disorders Unit and Massachusetts Alzheimer's Disease Research Center, Department of Neurology, Massachusetts General Hospital and Harvard Medical School, Boston, MA, USA

## Abstract

**Background:**

PPA refers to a clinical syndrome presenting with language impairment with relative preservation of other cognitive functions. Neuroimaging evidence suggests that the language network, anchored in left prefrontal and temporo‐parietal cortices, is selectively affected in PPA. To date, no pharmacological or neuromodulation strategies can satisfactorily improve symptoms in PPA. Focal neuromodulation techniques, such as repetitive TMS (rTMS), can change resting‐state functional connectivity in a network‐specific manner. Thus, we investigated whether rTMS could selectively modulate functional connections within the degenerated language network in PPA.

**Method:**

A double‐blinded, sham controlled, cross‐over design was used to administer intermittent theta burst stimulation (iTBS) for 10 days in a heterogeneous sample of PPA patients: 4 logopenic variant (lvPPA), 2 non‐fluent variant (nfvPPA), 1 semantic variant (svPPA), and 3 with primary progressive apraxia of speech (PPAOS). We stimulated the left caudal middle frontal gyrus region most functionally correlated with the language network on an individual‐subject basis. Standardized language and functional communication assessments were administered at baseline, post‐active TMS, and post‐sham TMS. Resting‐state fMRI data were analyzed to probe functional connectivity changes across sessions.

**Result:**

We found language improvements selective to active TMS in the functional communication analysis and standardized language assessment in all PPA subtypes and PPAOS. Across all subtypes, increased functional connectivity was observed in the language and other networks subserving cognitive performance after active TMS.

**Conclusion:**

We demonstrate preliminary evidence that personalized functional‐network guided iTBS can improve language impairments in a heterogeneous PPA and PPAOS cohort. Furthermore, improvements in language tests were accompanied by increases in functional network connectivity, pointing to a putative neural mechanism of TMS‐induced benefits in PPA and PPAOS.

